# Cloud-native encryption as a service for IoT

**DOI:** 10.1038/s41598-026-52815-x

**Published:** 2026-07-15

**Authors:** Amir Javadpour, Tarik Taleb, Chafika Benzaid, Khaled Zeraoulia, Abderezzak Djebrani, Mohamed Yacine Belhadi, Luis Cordeiro, Luís Rosa

**Affiliations:** 1 Senior Cybersecurity Researcher MOSA!C Lab / ICTFICIAL Oy, Espoo, Finland; 2 Faculty of Electrical Engineering and Information Technology, Ruhr University Bochum, Bochum, Germany; 3 Faculty of Information Technology and Electrical Engineering, University of Oulu, Oulu, Finland; 4 Houari Boumediene University of Science and Technology (USTHB), Bab Ezzouar, Algeria; 5 OneSource, 3030-384 Coimbra, Portugal

**Keywords:** Encryption as a service (EaaS), Internet of things (IoT), Kubernetes, Cloud-native security, Fog computing, Edge computing, Key management, Multi-tenant isolation, Microservices, Secure service deployment, Engineering, Mathematics and computing

## Abstract

Encryption as a Service (EaaS) is a practical solution for resource-constrained Internet of Things (IoT) devices that cannot efficiently execute costly cryptographic tasks locally. This paper presents a cloud-native EaaS platform implemented on Kubernetes and designed to support scalable encryption, decryption, and key-management services for IoT environments. The paper describes the functional architecture of the platform, defines its main service workflow, and introduces two deployment modes, namely cloud-based and fog-based deployment. The proposed platform is evaluated in terms of processing time, deployment time, and end-to-end response time. The results show that the fog-based deployment reduces the response time by at least $16\%$ for small payloads and by up to $6.8\times$ for larger payloads compared with the cloud-based mode. The deployment analysis also shows that increasing the number of replicas from 1 to 5 leads to a deployment-time increase of more than $30\%$, while increasing the workload to 11 replicas results in an increase of about $47\%$. In addition, the results indicate that the Key Manager is the most resource-intensive component and has the highest impact on pod readiness time. Overall, the findings show that the proposed Kubernetes-based EaaS platform can provide flexible and scalable cryptographic support for IoT systems, while fog-based placement offers clear latency advantages in the evaluated prototype setting.

## Introduction

Encryption as a Service (EaaS) is the process of providing all cryptographic services to the end users, and it overcomes the resource limitation issues of the end devices. With EaaS, organizations can outsource the complex and time-consuming tasks of encryption and key management to third-party providers who specialize in these processes. This allows them to focus on their core business operations while maintaining the security and integrity of their sensitive data^1–3^. EaaS offers numerous benefits, including easy implementation and scalability, reduced costs of ownership and maintenance, and enhanced security measures^4–6^.

One of the needful users for EaaS solutions is the Internet of Things (IoT) devices. They have limited resources, and due to their large scale and the popularity of such networks among modern digital societies, a scalable EaaS framework is needed^7,8^. However, managing and maintaining an EaaS platform for this demand is challenging^9^.

Some research on EaaS has been conducted during the current decade.Al-Tamimi et al.^10^ have analyzed the performance of implementing three symmetric cryptographic algorithms on an EaaS platform. This research aims to give insights into the performance of these algorithms to be deployed in real-world environments. Ibtihal et al., El Bouchti et al.^11,12^ have proposed an EaaS platform deployed on cloud servers, where users can request for encrypting/decryption images. The servers are deployed on OpenStack, and the Paillier encryption algorithm is served. Another fog-based EaaS is proposed by Deb et al.^13^, where heterogeneous IoT devices are served with cryptographic services based on their demands, the available resources, and network conditions.Ihtesham et al.^14^ have proposed a searchable EaaS platform to preserve users’ privacy. This platform is deployed on the Contabo public cloud. The reviewed research does not focus on the scalability of the deployed EaaS solution. Another EaaS solution, the closest to our work, is proposed by Merdan et al.^15^; the main goals are to be scalable, extendible, easy to deploy, and deployed on Kubernetes. The limitation of this work is that it does not provide adequate details for implementing their proposed solution and lacks analysis of different architectures.

As a result, this paper proposes a new cloud-native EaaS platform and provides two methods for its deployment. The proposed platform is implemented on Kubernetes to facilitate the process of auto-scaling, maintenance, and data retrieval^16^. The paper significantly contributes to the EaaS field by proposing a novel cloud-native EaaS platform implemented on Kubernetes. Firstly, it presents an architecture for the EaaS solution, detailing the structural framework and how various components interact within the platform. It also suggests two distinct deployment methods for the EaaS platform, offering users flexibility in choosing deployment approaches that suit their specific needs and preferences. The paper provides detailed implementation guidelines for deploying the EaaS framework on Kubernetes, ensuring scalability and effective maintenance. The proposed cloud-native EaaS platform on Kubernetes aims to provide flexible, scalable, and efficient cryptographic solutions for modern computing environments. It addresses the challenges of traditional encryption methods, which can be resource-intensive and complex to manage in dynamic and distributed systems^17^. The key contributions of this paper are:

- Proposing the architecture of a cloud-native EaaS solution.
- Suggesting two different deployment methods and discussing their benefits.
- Providing the details of implementing the proposed framework on Kubernetes to make it scalable and well-maintained.

These contributions collectively advance the understanding and adoption of cloud-native EaaS solutions, paving the way for enhanced security capabilities in various domains.

The remainder of this paper is as follows. Section Related Work and Research Gap reviews the most relevant studies and identifies the main research gap. Section Proposed EaaS Architecture presents the functional architecture of the proposed EaaS platform and describes its main components and workflows. Two deployment methods, cloud-based and fog-based, are introduced in Section Proposed Deployment Methods, and the advantages of each are discussed. These deployment methods are designed to help organizations choose the most suitable option based on their service requirements and available resources. In Section Evaluation Results, the results for different timing metrics are analyzed. Finally, Section Conclusion summarizes the paper and presents the main conclusions.

## Related work and research gap

Encryption as a Service (EaaS) has become an important solution for devices with limited resources. This is especially true for Internet of Things (IoT) devices, which often have limited processing power, memory, and energy. In such settings, sending heavy cryptographic tasks to an external service can reduce the burden on end devices and improve security management^1,3,7^. At the same time, the service platform itself must be scalable, easy to manage, and reliable when many users connect at the same time^8,9^.

Several studies have already explored EaaS from different viewpoints. Early works mainly focused on cloud-based encryption services. For example^12^, proposed an encryption service for healthcare cloud security, while^11^ studied homomorphic encryption as a service for outsourced image processing. These studies show the practical value of EaaS, but they mainly focus on one use case and do not provide a complete cloud-native system design for large-scale IoT deployment.

Other studies have focused on performance or service extensions. The work in^10^ evaluated the runtime performance of several encryption algorithms in an EaaS setting. The CEaaS framework in^13^moved the discussion toward fog-enabled IoT and considered constrained resources and network conditions. In addition^14^, proposed a privacy-preserving searchable encryption service. These works improve important parts of the service, but they do not fully address platform architecture, practical orchestration, and deployment trade-offs for a cloud-native EaaS platform (Table 1).

**Table 1:** Comparison of related studies and the proposed work.

Work	Main focus	Deployment	Scalability	Security/service scope	Main limitation
^11,12^	Cloud-based encryption service	Cloud	Limited	Data and image protection	Focus on specific use cases; no cloud-native deployment analysis
^10^	Performance evaluation of EaaS algorithms	Service-level study	Not central	Runtime comparison	Focus on algorithm performance only
^13^	Constrained EaaS for fog-enabled IoT	Fog/IoT	Partial	Resource-aware cryptographic service	Limited cloud-native orchestration detail
^14^	Searchable and privacy-preserving EaaS	Cloud	Partial	Searchable encryption	Focus on service function, not full deployment design
^15^	Dockerized cryptography-as-a-service	Containerized	Yes	Multi-purpose cryptographic framework	Limited analysis of deployment choices
^18^	Cloud-native EaaS architecture	Cloud-native	Yes	Architecture-level service design	Limited comparison of practical deployment methods
^17^	Review of EaaS architectures and taxonomies	Review	N/A	Literature organization	Does not provide implementation and evaluation
^19^	Kubernetes deployment of an EaaS testbed	Kubernetes	Yes	Practical deployment	Limited architecture comparison in one unified platform
^20^	Full-cloud-fog EaaS architecture	Cloud + fog	Yes	Throughput and security improvement	Different focus from detailed component-level deployment analysis in this paper
This work	Cloud-native EaaS platform with two deployment methods	Kubernetes, cloud-based and fog-based	Yes	End-to-end service workflow and timing evaluation	—

There are also studies that are closer to practical deployment. The work in^15^ introduced a dockerized and multi-purpose cryptography-as-a-service framework with attention to scalability and extensibility. The work in^18^ also discussed an encryption-as-a-service architecture on a cloud-native platform. In addition, the recent review in^17^ organized the EaaS literature based on architectures and taxonomies. More recent studies have further moved the field toward implementation and deployment by studying Kubernetes-based EaaS deployment and a full-cloud-fog EaaS architecture^19,20^. These studies are very relevant to our work because they show that EaaS is moving from basic service models toward deployable and scalable cloud-native systems.

At the cloud-native level, Kubernetes-based systems also require secure and manageable operation. Existing studies have highlighted issues such as scheduling, load balancing, secret handling, and scalable security management in Kubernetes environments^9,16,21,22^. In addition, recent guidance on API protection for cloud-native systems shows that secure API exposure and control are necessary for production-grade service platforms^23^. These points are important for EaaS because the platform is not only an encryption engine, but also a service system with interfaces, orchestration logic, and management functions.

Based on the reviewed literature, three main gaps can be identified. First, many existing studies focus on encryption methods or specific service functions, but not on a complete cloud-native EaaS architecture. Second, some studies discuss scalability, but they provide limited details on practical deployment options and component interaction on Kubernetes. Third, the difference between cloud-based and fog-based deployment is still not discussed in a clear and structured way for this kind of platform. Therefore, this paper proposes a cloud-native EaaS architecture on Kubernetes, presents two deployment methods, and evaluates them using processing time, deployment time, and response time.

## Proposed EaaS architecture

This section describes the proposed EaaS architecture, its components, and how they interact.

**Fig. 1:**
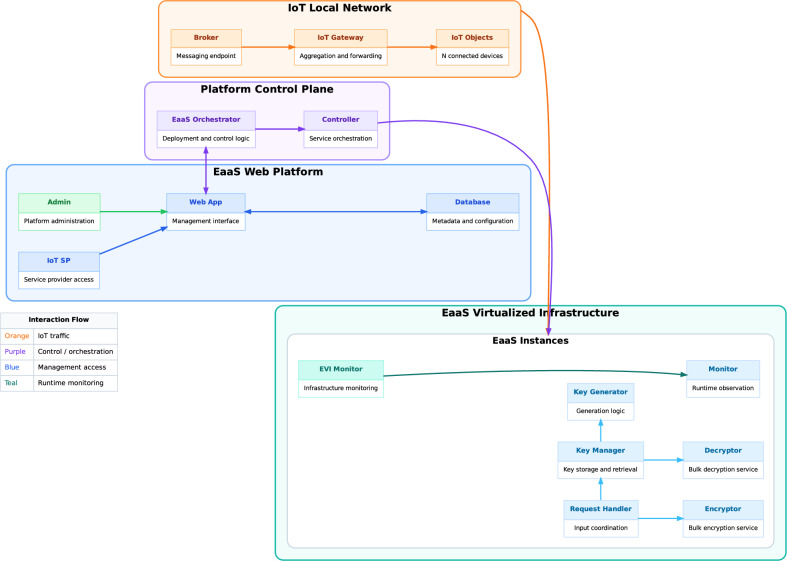
The proposed EaaS architecture.

The proposed architecture, illustrated in Figure 1, comprises four components: IoT network, web application, EaaS orchestrator, and virtualized infrastructure. The IoT network contains the end devices/objects connecting to the platform for cryptographic services. They send their messages to the gateway through a broker. These devices’ access to the services is allowed after the IoT service provider (IoTSP) subscribes to the platform, specifies the required services, and provides information about its local network using the web application. The platform administrator can also connect to the platform through the web application to monitor and manage the whole platform. The web application is an entry point to the platform that facilitates and automates the service management procedures. On the other hand, the EaaS orchestrator transfers the administrator’s and the IoTSP’s requests to the infrastructure and is also responsible for providing data retrieval and seamless maintenance services.

The proposed architecture comprises a virtualized infrastructure with a controller and multiple instances. The controller is critical in managing connections between instances to ensure users receive the necessary services. The controller is the heart of our platform; it manages the connections between the cases to ensure that end users are served based on their requests. The flow of data between different instances is under the controller’s supervision. Now, let us dive into the functionalities of an EaaS instance and the workflow of procedures our platform supports.

### Security boundary, key lifecycle, and transport assumptions

The platform description makes the trust boundary explicit. IoT devices authenticate to the platform through mutual TLS (mTLS). In our design, the external mTLS session terminates at the Request Handler service boundary, and the request is then forwarded to the internal services over mTLS-protected service-to-service channels. Therefore, the bulk data for the requested operation are not assumed to be already encrypted end-to-end for that same operation. Instead, the Request Handler receives the protected request, validates the tenant and device identity, and forwards the plaintext or ciphertext only for the time needed to complete the requested cryptographic task. To reduce exposure, sensitive payload logging is disabled, plaintext is kept only in memory, and temporary buffers are cleared after the operation finishes^23,24^.

Long-term tenant keys are not stored in plaintext in PostgreSQL. The database stores only wrapped key blobs and key metadata, including tenant and device binding, key version, creation time, expiry time, and revocation state. The wrapping key stays outside the database boundary and is assumed to be protected by an external KMS or HSM through envelope encryption^22,25^. For actual encryption and decryption requests, the Key Manager releases only a short-lived key handle or a wrapped per-request data-encryption key to the target service. As a result, persistent tenant keys do not move in plaintext between microservices.

Table 2 summarizes the complete lifecycle of the key material. Key creation happens when a tenant is provisioned or when a device first requests an algorithm-specific key. Key rotation is supported by versioned key identifiers and can be triggered periodically or immediately after a suspected compromise. Revocation disables future operations for the affected version, while deletion removes expired wrapped blobs and related metadata after the retention period. Access to the repository is logged per tenant, device, service account, operation type, and timestamp to support audit and incident response. If a database dump or backup is disclosed, the attacker only obtains wrapped key material and metadata; the impact is contained per tenant because each tenant uses a separate key hierarchy and namespace-scoped service access.

**Table 2:** Key lifecycle and repository protection in the EaaS design.

Phase	Stored artifact	Protection	Trigger	Containment/audit action
Creation	Tenant root key version, device binding metadata, wrapped seed or DEK template	Envelope encryption with external KMS/HSM; metadata in PostgreSQL	Tenant onboarding or first valid request	Audit record created for tenant, device, algorithm, and service account
Storage	Wrapped key blob, version, status, expiry, tenant ID	Database stores wrapped material only; access is RBAC-limited and logged	Normal operation	Namespace and tenant scoping limit cross-tenant exposure
Rotation	New key version, old version marked retiring	Versioned replacement; old key kept only for bounded decryption window	Periodic schedule or incident response	Rotation event logged; new operations use only latest active version
Revocation	Revoked status flag and deny rule	Key lookup rejects revoked versions	Device loss, tenant request, or suspected compromise	Revocation blocks future use and creates an audit alert
Deletion	Removal of expired wrapped blob and metadata	Secure cleanup after retention period	Expiry, tenant offboarding, or compliance rule	Deletion event logged; backup restoration still requires KMS/HSM access

### The instances

The EaaS virtual instances perform the key functionalities of our proposed platform. They provide cryptographic services, such as encryption, decryption, and key management. Separate instances are assigned to different IoTSPs once they subscribe to the platform. This separation helps in customizing their requested services and also provides a sort of isolation. An EaaS virtual instance is composed of the following modules:

**Algorithm 1:**
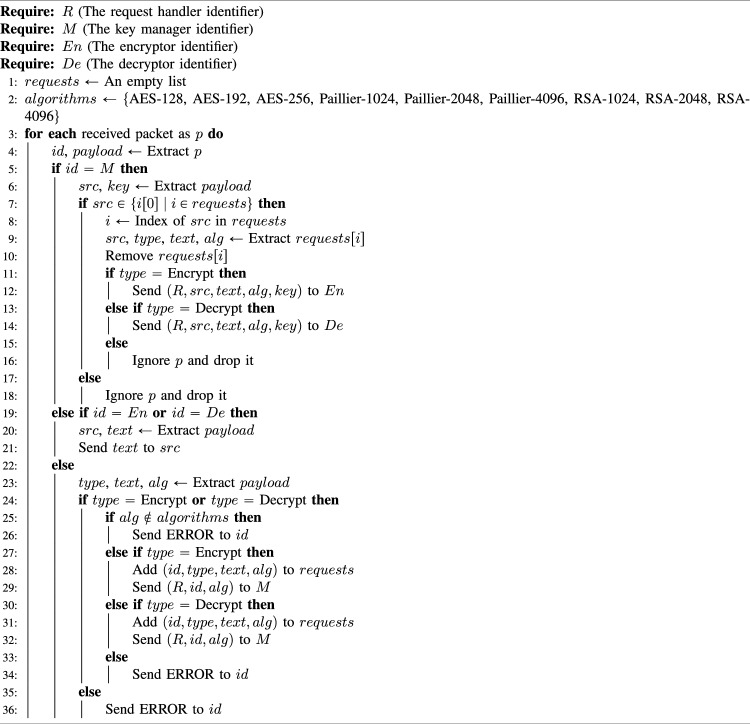
The procedure of the request handler module

**Algorithm 2:**
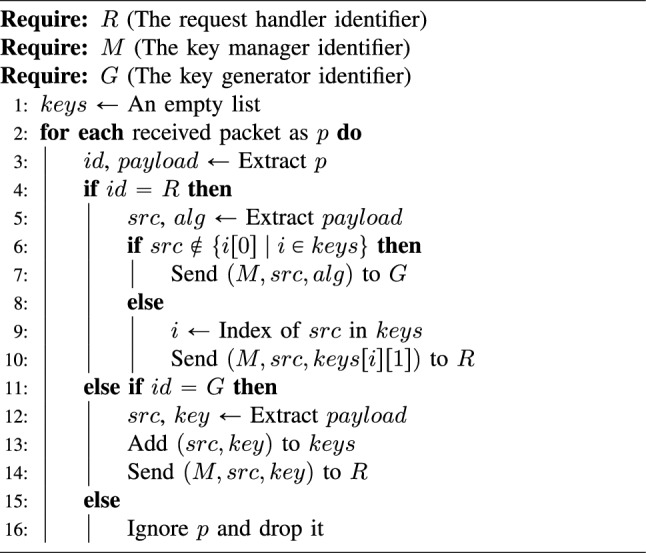
The procedure of the key manager module

**Algorithm 3:**
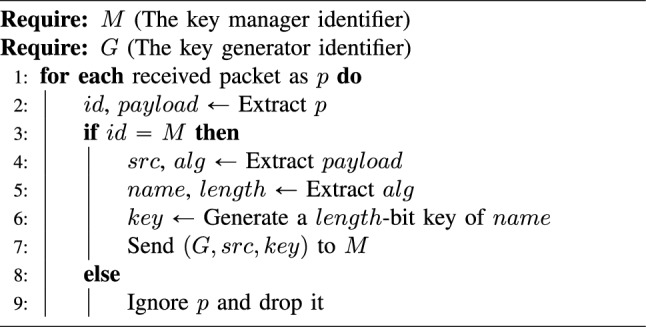
The procedure of the key generator module

**Algorithm 4:**
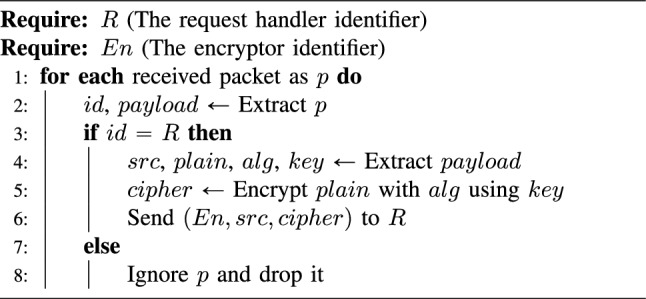
The procedure of the encryptor module

**Algorithm 5:**
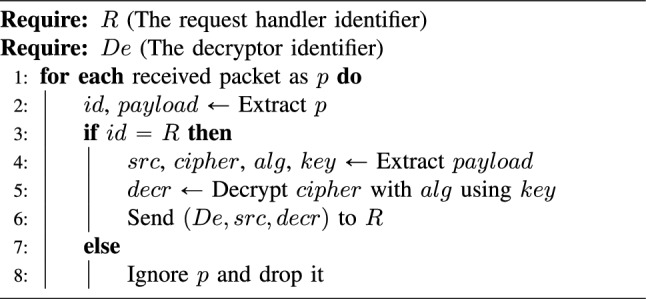
The procedure of the decryptor module

- Request handler: It is responsible for communicating with the IoT devices. When they send their request, the request handler checks its validity, stores it with the identifier of the requester device, and forwards it to the key manager module. On the way back, the request handler forwards the received encrypted/decrypted data from the platform modules to the related IoT device. The algorithm that describes this module in detail is presented in Algorithm 1. Algorithm 1 starts by initializing some variables and data structures, such as an empty list for storing requests and a set of supported encryption algorithms. Then, it enters a loop to handle incoming packets. Each packet is examined based on its identifier (*id*) to determine its type and origin. For the Key Management, If the packet is from the key manager (*M*), it extracts the source (*src*) and key information. If the source is already in the list of pending requests, it processes the request accordingly (either encryption or decryption) and sends the corresponding data to the encryptor (*En*) or decryptor (*De*). For the encryption/decryption process, if the packet is from the encryptor or decryptor, it sends the decrypted/encrypted text back to the source. In error handling, If the packet does not match any expected format or has an unsupported encryption algorithm, it sends an error message to the appropriate entity. Then, In packet ignoring, If the packet does not match any expected identifier, it is ignored and dropped. After that, in loop continuation, for each packet, the loop continues until all received packets are processed.
- Key Manager: As its name implies, this module manages the encryption/decryption keys. If the related keys are not in the database, the key manager asks the key generator module to create new ones and then sends them to the request handler module. The algorithm of this module is shown in Algorithm 2. Algorithm 2 presents the functionality of the Key Manager module. It facilitates generating and distributing encryption keys to request handler modules, ensuring secure encryption and decryption operations. Upon receiving packets, the algorithm processes requests from request handlers to obtain encryption keys. If a key has not yet been generated, the Key Generator module is triggered to create one. Otherwise, it retrieves the existing key and forwards it to the appropriate module. This streamlined key management process enhances the security and efficiency of the encryption system.
- Key Generator: This module can generate the required encryption/decryption keys with different sizes and based on different algorithms. When new keys are generated, they are added to the Key Mapper database by the key manager module. The process done by the key generator module is presented in Algorithm 3. Algorithm 3 describes the procedure of the Key Generator module within the system. The Key Generator module receives packets and extracts the identifier *id* and payload *payload* from each packet. If the identifier matches the key manager identifier *M*, indicating a request for key generation, the algorithm further extracts the source *src* and encryption algorithm *alg* from the payload. It then generates a key of the specified length *length* and algorithm *name*, and sends the generated key back to the key manager module *M* along with the source *src*. If the identifier does not match *M*, the algorithm ignores and drops the packet. The Key Generator module efficiently handles requests for key generation, contributing to the secure encryption operations within the system.
- Encryptor: The process of getting the plaintext, encrypting it with the provided key, and generating the ciphertext is done by this module. We can see a detailed description of this module in Algorithm 4. Algorithm 4 is the procedure of the Encryptor module within the system. The Encryptor module receives packets and extracts the identifier *id* and payload *payload* from each packet. If the identifier matches the request handler identifier *requestHandler*, indicating a request for encryption, the algorithm further extracts the source *src*, plaintext *plain*, encryption algorithm *alg*, and encryption key *key* from the payload. It then encrypts the plaintext *plain* using the specified encryption algorithm *alg* and encryption key *key* to generate the ciphertext *cipher*. and then, it sends the ciphertext *cipher* and the source *src* to the request handler module *R*. If the identifier does not match *requestHandler*, the algorithm ignores and drops the packet. So, the Encryptor module plays a crucial role in encrypting plaintext data for secure transmission within the system.
- Decryptor: Similar to the encryptor module, the Decryptor module’s responsibility is to get the ciphertext, decrypt it with the provided key, and generate the decrypted text. This module is completely described in Algorithm 5. In Algorithm 5, The Decryptor module receives packets and extracts each packet’s identifier *id* and payload *payload*. If the identifier matches the request handler identifier *requestHandler*, indicating a decryption request, the algorithm further extracts the source *src*, ciphertext *cipher*, encryption algorithm *alg*, and decryption key *key* from the payload. It then decrypts the ciphertext *cipher* using the specified encryption algorithm *alg* and decryption key *key* to obtain the decrypted plaintext *decr*. and, it sends the decrypted plaintext *decr* and the source *src* to the request handler module *R*. If the identifier does not match *requestHandler*, the algorithm disregards and discards the packet.
- Monitor: This module must fulfill two responsibilities: Collecting the metrics and logging the activities. It is worth noting that not all the IoTSPs ask for this module, and it is optional.

### Control-plane message format and failure handling

Algorithms 1–5 describe the logical message flow between services. In the implementation, each control-plane message uses the fixed header fields summarized in Table 3. Therefore, the identifiers *M*, *G*, *En*, and *De* represent authenticated service identities rather than unauthenticated raw packet labels. Peer authentication and message integrity are provided by mTLS between services, while request freshness is checked with a request identifier, a nonce, and a bounded timestamp window^23,24^. Messages that fail authentication, exceed the allowed clock skew, reuse a nonce, or exceed the timeout value are rejected and logged. Each tenant queue is also rate-limited to reduce request flooding and replay amplification.

**Table 3:** Control-plane message fields and robustness checks.

Field	Purpose	Validation rule
ver	Protocol version	Must match a supported service version
tenantID, deviceID	Tenant and device binding	Must match the authenticated client identity and namespace policy
reqID	End-to-end request correlation	Must be unique within the active replay window
op, alg	Requested operation and algorithm	Must belong to the allowed service profile
keyRef	Short-lived key handle or wrapped DEK reference	Must match an active key version and tenant scope
ts, ttl	Freshness and timeout control	Request is dropped if too old or expired
nonce	Replay protection	Duplicate nonce for the same device and time window is rejected
payloadLen	Size validation	Must stay below the configured limit for the service tier
status, errCode	Failure reporting	Returned on validation, timeout, or backend failure

If the Key Manager or Key Generator does not answer before the timeout, the Request Handler removes the pending record and returns an explicit error to the device. Similarly, stale requests are deleted from the pending list, and failed internal operations are not retried indefinitely. These additions make the protocol description stronger and clarify how forged replies, replay attempts, and queue growth are handled in practice.

### The workflows

Three different procedures are supported by our proposed platform, which are subscription, encryption, and decryption.

#### Subscription

When IoTSPs sign up for our platform, they can customize the cryptographic service related to their preferred algorithms in several required instances based on their network workload and service duration. Our platform allows them to scale their allocated resources easily, ensuring optimal payments while maintaining efficiency.

#### Encryption

**Fig. 2:**
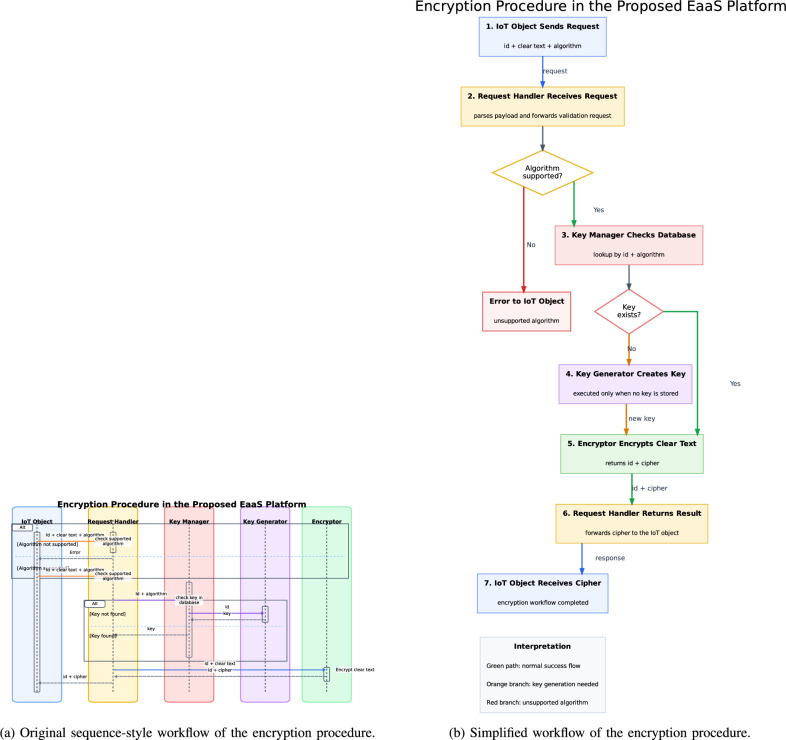
The workflow of the encryption procedure in the proposed EaaS platform. The left subfigure shows the original sequence-style interaction view, while the right subfigure presents a simplified and more readable Graphviz-based process view.

The encryption workflow is illustrated in Figure 2.

When the request handler receives a request from the IoT network, and the request type is “encrypt”, it first checks if the requested algorithm is supported by the instances available for the subscribed service. The request handler sends an error message to the related IoT device if it is not supported. Otherwise, the request handler sends the device identifier and the requested algorithm to the key manager. The key manager then checks if a valid key version corresponding to the specified identifier exists. If it exists, the key manager returns a short-lived key handle or wrapped per-request key reference to the request handler. If it does not exist, the key manager requests new material from the key generator. When the key material is generated, it is transmitted to the key manager, wrapped, stored with its metadata, and mapped to the device identifier before a short-lived reference is returned to the request handler. The device identifier, the plaintext, and the short-lived key reference are then sent to the encryptor through the request handler. The encryptor resolves the operation-specific key in memory, encrypts the plaintext, and returns the ciphertext to the request handler. Finally, the ciphertext is sent to the related IoT device through the request handler. In this design, the secure client channel terminates at the Request Handler boundary, while internal service-to-service communication is also protected by mTLS.

#### Decryption

**Fig. 3:**
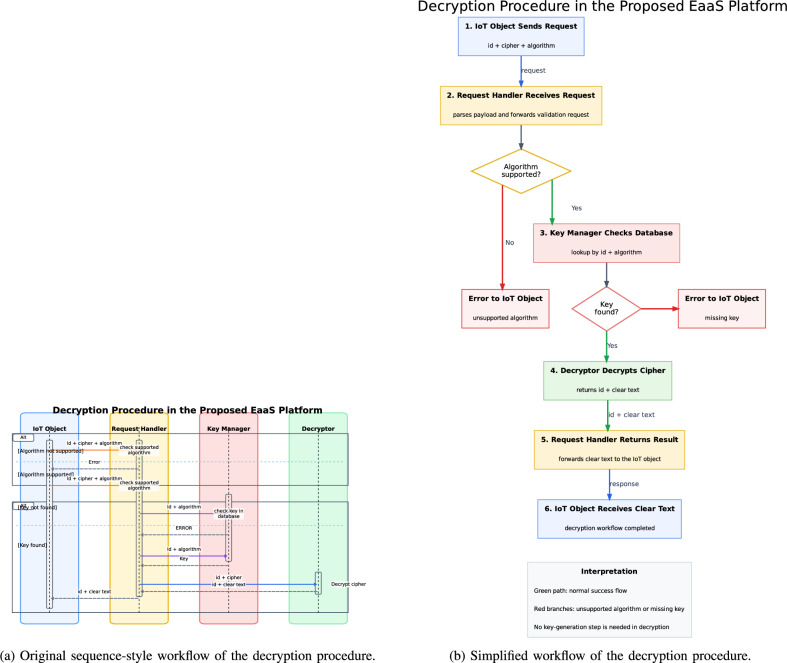
The workflow of the decryption procedure in the proposed EaaS platform. The left subfigure shows the original sequence-style interaction view, while the right subfigure presents a simplified and more readable Graphviz-based process view.

The decryption workflow is shown in Figure 3.

The decryption workflow begins by providing the request handler with the necessary data, such as the device identifier, the ciphertext, the algorithm type, and the request type, which is “decrypt” here. Upon receiving it, the request handler checks if the instances of the current service support the algorithm. If not, an error message is returned to the related IoT device. If the algorithm is supported, the device identifier is forwarded to the key manager, which searches for the corresponding active key version. If the key is found, a short-lived key handle or wrapped per-request key reference is returned to the request handler. However, if it does not exist, the key manager informs the request handler that the device with that identifier must first generate the appropriate key(s). Subsequently, the request handler transmits the device identifier, the ciphertext, and the short-lived key reference to the decryptor. The decryptor then resolves the required key only in memory, decrypts the ciphertext, and returns the decrypted text to the request handler. At last, the request handler sends the extracted text back to the related IoT device^24^. This clarification makes the trust boundary explicit and avoids transferring persistent tenant keys in plaintext between modules.

## Proposed deployment methods

We suggest two distinct deployment methods: Cloud-based and Fog-based methods. Each IoTSP chooses one of these methods based on their requirements, budget, and preferences. The remaining section explores each method’s benefits and offers recommendations on the most suitable method for different situations.

### Cloud-based EaaS

**Fig. 4:**
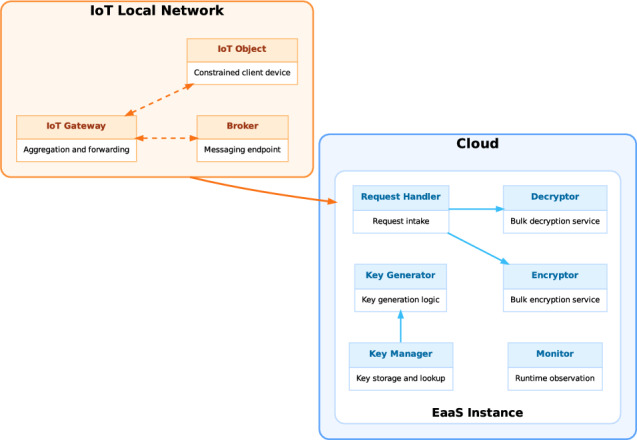
The location of different instances in the proposed cloud-based deployment.

All the infrastructure instances are on cloud hosts, as shown in Figure 4.

This deployment method allows for the centralized control and management of different instances, simplifying the processes of monitoring, maintaining, and updating automation. Furthermore, this centralization empowers robust security measures and ensures compliance with industry standards. Moreover, the IoTSPs experience adequate flexibility for scaling the resources up or down regarding their demand, optimizing the paid cost. Cloud-based deployments also offer advantageous features such as:

- *High availability:* Cloud deployments can distribute replicas across different nodes and restart failed pods automatically, which reduces service interruption and improves resilience.
- *Fast deployment:* Cloud-based deployments can be quickly set up and deployed with little to no hardware installation or configuration, allowing users to begin using the service fast and efficiently.
- *High performance:* The deployment can be adjusted for maximum performance, with quick and dependable access to computer resources, data, and services.
- *Fault tolerability and availability:* Since cloud environments have access to a large number of resources, they can easily handle faults and also replace unavailable resources with configurable ones.

### Fog-based EaaS

**Fig. 5:**
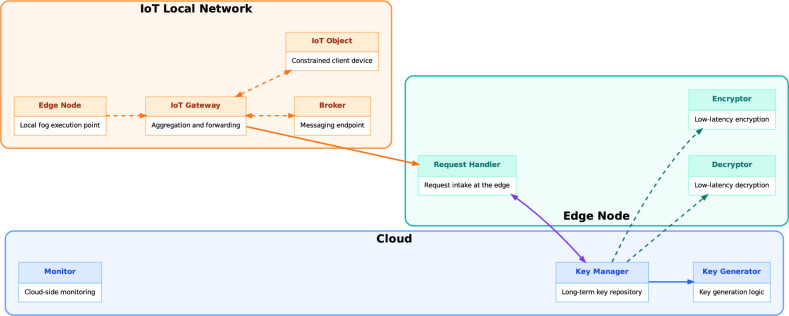
The location of different instances in the proposed fog-based deployment.

Unlike the cloud-based deployment method, not all the infrastructure instances are located on cloud hosts, and some are hosted by fog/edge nodes. As we can see in Figure 5, only the key manager, the key generator, and the monitoring modules are located on the cloud.

The request handler, the encryptor, and the decryptor modules are within the fog layer. This method distributes the processing load among cloud and fog nodes while keeping the workflows consistent. Fog-based deployment has its advantages, too. The fog nodes are closer to the end devices, and hence, they can reduce latency. This feature is more advantageous for situations where there are stringent end-to-end delay limitations. Processing the tasks close to end devices can also improve data privacy and security. The closer nodes are less exposed to potential leakages during the transmission phases. On the other hand, there are also some general benefits of fog-based deployments, such as: In the fog-based mode, only short-lived key handles or wrapped per-request keys travel between the cloud and the fog layer. The Request Handler at the fog side may keep a bounded in-memory cache of active key handles for a short TTL, and the cache is invalidated on key rotation, revocation, or expiry. Therefore, repeated requests do not require a cloud round trip for every operation, while long-term tenant keys remain inside the cloud-side key management boundary.

- *Low bandwidth consumption:* Only keys need to be transmitted from the cloud in edge-based deployments, which reduces the amount of data that needs to be transmitted over the network.
- *Lower cost:* Fog nodes can lead to lower operational costs because they require less data throughput and can rely less on cloud-based infrastructures.
- *Real-time processing:* Fog-based deployment methods enable real-time data processing and analysis, enabling customers to respond to real-time events and situations.

### Isolation and supply-chain controls

Namespace separation and RBAC are the base isolation mechanisms, but they are not the only protections used in the design. The platform also uses namespace-scoped NetworkPolicy rules, Pod Security Admission with the *restricted* profile, non-root containers, read-only root file systems, dropped Linux capabilities, seccomp *RuntimeDefault*, and tightly scoped service accounts^26–28^. PodSecurityPolicy is not used because it was deprecated and later removed from modern Kubernetes releases; for this reason, the manuscript now refers to Pod Security Admission and admission-policy enforcement instead^27,29^. Table 4 lists the protections that were enabled or assumed in the experimental setup description. The container supply chain is controlled through a simple CI/CD path. Images are built from version-controlled Dockerfiles, scanned before publication, accompanied by an SBOM, signed in CI, pushed only to the private registry, and admitted into the cluster only if the signature, digest policy, and namespace policy checks succeed^26,30^. This clarification strengthens the threat discussion for image tampering and deployment-time attacks.

**Table 4:** Isolation and supply-chain controls used by the platform description.

Control area	Mechanism	Purpose in this work
Tenant separation	One namespace per tenant instance, namespace quotas, RBAC	Limits cross-tenant access and narrows the blast radius of compromise
East-west traffic	Namespace-scoped NetworkPolicy and mTLS between services	Restricts service reachability and protects service-to-service traffic
Pod hardening	Pod Security Admission (*restricted*), non-root, read-only root FS, dropped capabilities, seccomp	Reduces runtime privilege and container escape risk
Service identity	Dedicated service accounts and least-privilege roles	Limits access to secrets, logs, and key metadata
Ingress/egress	Request Handler exposure only, internal services kept private	Narrows the external attack surface
Image integrity	Signed images, digest pinning, admission checks	Prevents unsigned or modified images from running
Supply-chain visibility	SBOM generation and vulnerability scanning in CI	Improves traceability and patch management
Audit trail	Access logs for keys, deployments, and policy denials	Supports incident response and tenant-level accountability

## Evaluation results

**Fig. 6:**
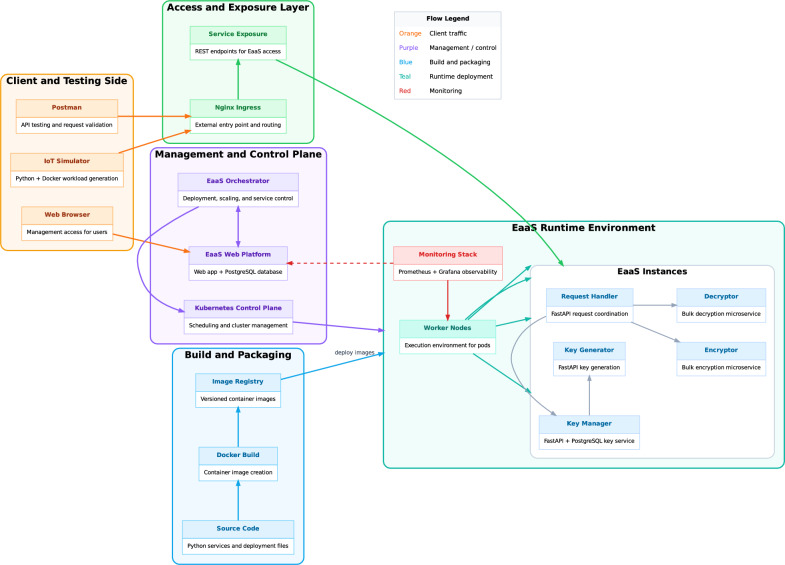
The development tools used for implementing the proposed framework.

To assess the performance of our work, we have implemented the proposed EaaS platform based on the two proposed deployment methods using open-source tools. Figure 6 shows the tools used for implementing our proposed framework.

The EaaS orchestrator and the web application are developed in Django on a single server. A PostgreSQL database is used to store the subscription data. Kubernetes serves the virtualized infrastructure, and its clusters are composed of a single master node as the controller and two worker nodes. The master node uses Prometheus for data retrieval and Grafana for data visualization^21,22^. The infrastructure instances are executed on Docker containers, which Uvicorn powers to serve FastAPI as the web framework. The key mapper module (i.e., the database for storing the cryptographic keys) is Postgres-based, and the request handler is exposed to the IoT devices through an Nginx Ingress service. Finally, Docker containers running Python scripts simulate the IoT devices, and Postman is employed for API testing.

The current prototype focuses on a controlled platform evaluation rather than a wide-area network emulation study. Therefore, the IoT simulator, cloud services, and fog services were deployed in the same Kubernetes environment, and the reported response times mainly reflect application processing, service placement, and container routing inside the prototype. They do not include a calibrated WAN delay, jitter model, or bandwidth cap between the fog and cloud layers. This clarification is important for interpreting the measured gap between the cloud-based and fog-based modes.

In the fog-based mode, the cloud-located Key Manager does not return a long-term tenant key for every operation. Instead, it returns a short-lived key handle or wrapped per-request key, and the edge-side Request Handler may cache this handle for a bounded TTL. For repeated requests within the TTL window, the fog path therefore avoids a cloud round trip for every operation. This design choice explains why the fog deployment can still show lower response time even though the root key boundary remains in the cloud.

We have compared standalone IoT devices, i.e., those that perform cryptographic tasks by their processors, and the EaaS-aided IoT devices to evaluate the performance of our proposed framework. The simulated IoT devices have limited resources, such as 0.2 CPU units and 20*MB* of RAM, and they consist of two types: data generators and data readers. Data generators must encrypt their data, while the readers ask for decryption.

### Processing time

One of the metrics we have to measure is the processing time, which includes key generation, encryption, and decryption time in our framework.

**Fig. 7:**
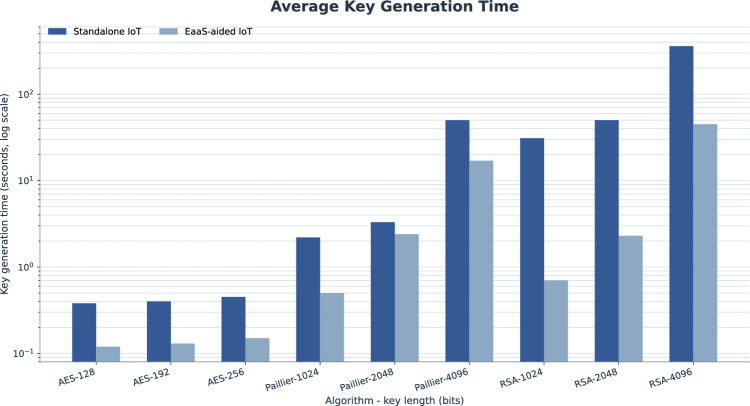
Comparing the average key generation time for standalone and EaaS-aided IoT devices in the proposed EaaS framework.

**Fig. 8:**
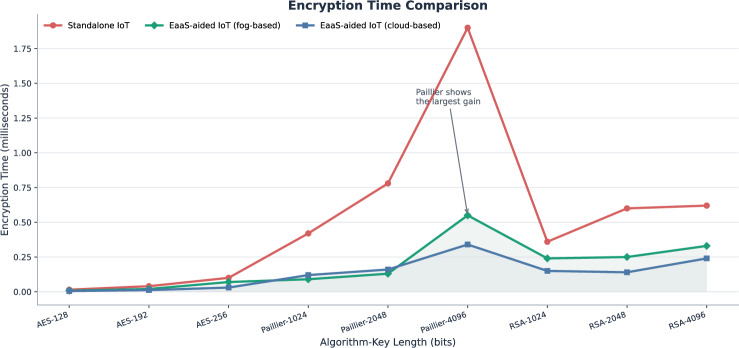
Comparing the average encryption time for standalone and EaaS-aided, cloud-based, and fog-based, IoT devices in the proposed EaaS framework.

**Fig. 9:**
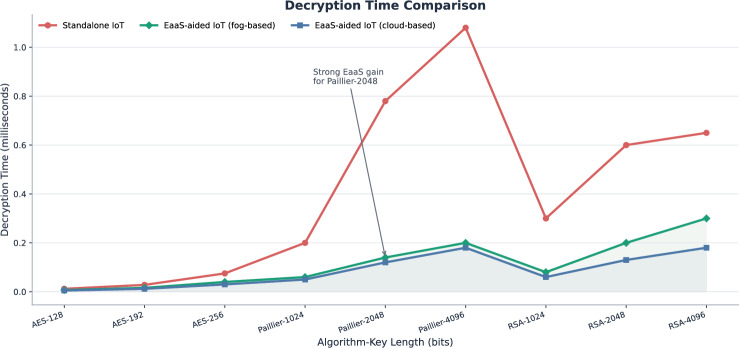
Comparing the average decryption time for standalone and EaaS-aided, cloud-based, and fog-based, IoT devices in the proposed EaaS framework.

- *Key generation time:* This metric is defined as the average time required for generating cryptographic keys, and it determines the performance of the key generation process. Lower key generation time indicates a more efficient approach. This metric is calculated based on Equation 1, where *K* is the total number of generated keys and $k_i$ and $\tilde{k}_i$ are the starting and ending time of generating the $i^{th}$ key, respectively.
  1 $$\begin{aligned} \text {Key Generation Time} = \dfrac{\sum \limits _{i = 1}^{K} \tilde{k}_i - k_i}{K} \end{aligned}$$
  The results regarding the key generation time are reported in Figure 7. This plot shows that the key length has the minimum impact on the key generation time for the AES algorithm, especially when the EaaS solution is utilized. In other words, the key generation time is almost identical across different sizes of AES keys, and even though the EaaS framework generates the AES keys three times faster than the standalone devices, the difference is only around 270 milliseconds. However, the resource limitation of IoT devices makes the key generation process slower, even for lightweight algorithms such as AES. Moving to the Paillier algorithm, significant performance differences become apparent. The standalone devices experience a substantial increase in the key generation time, ranging from approximately 220% between 1024-bit and 2048-bit keys to a 650% increase from 2048-bit to 4096-bit keys. On the other hand, the gaps between the key generation times of the standalone devices and EaaS-aided ones are also notable. For the 1024-bit key length, the proposed EaaS framework outperforms standalone devices by 80% with a 2-second gap. The performance increase is 70% with a 5-second gap for the 2048-bit key length and 70% with about a 40-second gap for the 4096-bit key length. These differences are attributed to the Paillier algorithm’s more complex mathematical operations during key generation and the limited resources of IoT devices struggling with computational demands for larger key sizes. In the case of RSA, significant performance variations are again observed. Standalone devices experience at least a 200% increase in key generation time each time the key size is doubled, while EaaS shows a 700% increase. Despite EaaS having a significantly larger margin of increase, it remains at least eight times faster than standalone approaches, reaching up to 44 times faster for the 1024-bit keys. This can be attributed to the RSA algorithm’s computationally intensive mathematical operations during key generation, resulting in longer time requirements as the key size increases. IoT devices’ limited resources may struggle further in handling these computational demands. It is worth noting that when comparing the key generation times across algorithms, AES demonstrates the fastest performance, followed by Paillier, and then RSA.
- *Encryption time:* Encryption time refers to the average duration of encrypting data items. Lower encryption time indicates a more efficient approach. Encryption time is defined as Equation 2, where *E* is the total number of plaintext or data items that are encrypted and $e_i$ and $\tilde{e}_i$ are the starting and ending time of encrypting the $i^{th}$ item.
  2 $$\begin{aligned} \text {Encryption Time} = \dfrac{\sum \limits _{i = 1}^{E} \tilde{e}_i - e_i}{E} \end{aligned}$$
  The comparison between the encryption time of different scenarios is shown in Figure 8. The first point about the results is that all three algorithms become slower with the increase in key size. Among them, since its computational complexity is more dependent on the key size, Paillier slows down higher than the others by about five times. The most important conclusion of these results is about the improvement our EaaS framework brings. They claim that, compared to the standalone approach, the EaaS solution reduces the encryption time by $38\%$, $84\%$, and $63\%$ for AES, Paillier, and RSA, respectively. The improvement is marginally significant for AES as it is considered a lightweight encryption algorithm compared to RSA and Paillier. In contrast, the RSA and the Paillier algorithms demonstrate substantial performance improvements with the EaaS solution. The largest performance gap can be seen for the Paillier algorithm provided by EaaS, which has an encryption process seven times faster than that of standalone devices. It is also worth noting that, regarding encryption time, the fastest algorithm is AES, followed by RSA, and then Paillier.
- *Decryption time:* Similar to the definition of encryption time, decryption time is the average time required for decrypting a set of data items. Lower decryption time indicates a more efficient approach. Decryption time is defined as Equation 3, where *D* is the total number of ciphertext or data items that are decrypted and $d_i$ and $\tilde{d}_i$ are the starting and ending time of decrypting the $i^{th}$ item.
  3 $$\begin{aligned} \text {Decryption Time} = \dfrac{\sum \limits _{i = 1}^{D} \tilde{d}_i - d_i}{D} \end{aligned}$$
  Figure 9 shows the time required for decrypting data items in different scenarios. Upon data analysis, we can make several conclusions about the decryption times. First, the effect of increasing the key length on decryption is comparable. All algorithms take longer to decrypt data when the key size is larger because they involve more intricate mathematical operations. Furthermore, for every increase in key size, there is a rise in the decryption time. This rise is about 60%, 130%, and 55% for AES, Paillier, and RSA, respectively. During the decryption process, the RSA and Paillier algorithms need computationally demanding mathematical processes, which causes greater noticeable slowdowns than AES. Additionally, although the difference is insignificant, the EaaS solution for the AES algorithm shows better decryption timings (i.e., about 23%) than standalone approaches across a range of key lengths. On the other hand, the EaaS solution shows notable gains in Paillier and RSA (i.e., 81% and 76%). The EaaS solution notably boosts decryption speed for the Paillier algorithm with a key length of 2048 bits, which is nine times faster than the standalone approach. These values signify noteworthy gains in efficiency. The different decryption speeds may be due to the limited resources of standalone devices, which may limit the decryption process, resulting in slightly slower decryption times than the EaaS solution. This difference is greater for the Paillier algorithm because of its homomorphic nature. Lastly, the order of fastest to the slowest algorithm for decryption remains the same as for encryption: AES, RSA, and then Paillier.

### Deployment time

Deployment time is one of the evaluation metrics that must be measured to address the trade-off between the overhead of the proposed framework and its benefits. A lower deployment time is preferable. In the manuscript, deployment time is defined as the time required until the last pod in a deployment cycle becomes ready. Accordingly, this metric is expressed as the maximum readiness interval among all deployed pods. More precisely, deployment time is defined in Equation 5, where *P* denotes the total number of deployed pods, and $p_i$ and $\tilde{p}_i$ represent the initiation and operational times of the $i^{\textrm{th}}$ pod, respectively.

4 $$\begin{aligned} T_{\textrm{deploy}}=\max _{1 \le i \le P}\left( \tilde{p}_i-p_i\right) \end{aligned}$$

5 $$\begin{aligned} \text {Deployment Time} = \max \limits _{1 \le i \le P} (\tilde{p}_i - p_i) \end{aligned}$$

**Fig. 10:**
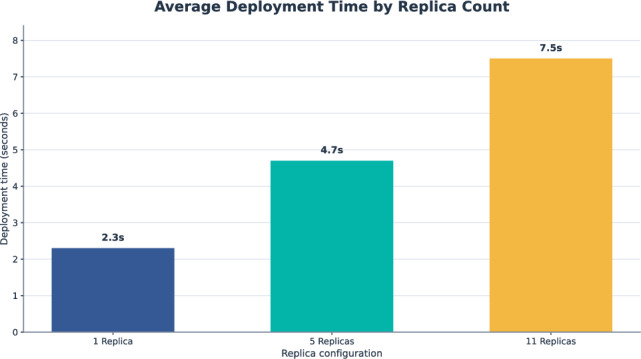
Comparing the average deployment time for different numbers of replicas that are used by the proposed EaaS framework.

**Fig. 11:**
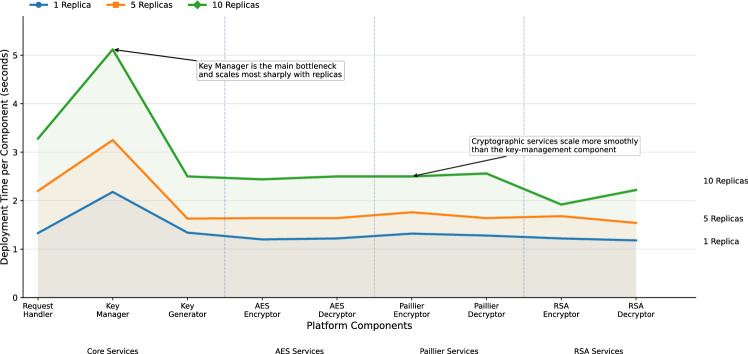
Comparing the deployment time per component for different numbers of replicas used by the proposed EaaS framework.

The per-component discussion in Figure 11 refers to the average readiness time of pods of the same component type and should not be confused with the overall deployment completion time. Figure 10 reports the deployment time of the scenarios with one, five, and eleven replicas per component.

In other words, these scenarios have 9, 45, and 99 pods, respectively. More detailed results are shown in Figure 11.

It is clear from examining the displayed results that extending the number of pods causes the deployment time to grow gradually. For example, the deployment time increases over $30\%$ when we increase the number of pods by a factor of 4, going from 1 replica per pod to 5 replicas. Similar to the previous example, a $120\%$ increase in workload (i.e., 11 replicates per pod) results in a 47% increase in deployment time. Different pod types have varying deployment times when looking at the deployment times per component. Interestingly, since the key manager exhibits the largest deployment time, there is a substantial correlation between the deployment time and component type.

It is evident that the EaaS framework effectively controls resource deployment and can handle a significant workload without requiring a notable increase in time. The differences in launch times can be explained by the fact that every pod has different requirements and dependencies regarding base images, libraries, and configurations. It takes longer to bootstrap the key management pod because it uses the greatest resources. Also, Integrating a cloud-native platform into the EaaS framework brings benefits such as scalability, resource efficiency, fault tolerance, and support for modern software development practices. Cloud-native platforms like Kubernetes provide automatic scaling and fault tolerance capabilities to handle fluctuations in workload and ensure the framework remains operational even in the face of failures.

### Response time

One of the main metrics that directly affects the quality of service is the response time. It is calculated by measuring the time between the initiation of a request and the reception of its response and then averaging this interval over all requests. In Equation 6, *R* is the total number of requests, $r_i$ is the sending time of the $i^{th}$ request, and $\tilde{r}_i$ is the receiving time of its response.

6 $$\begin{aligned} \text {Response Time} = \dfrac{1}{R}\sum \limits _{i = 1}^{R} (\tilde{r}_i - r_i) \end{aligned}$$

**Fig. 12:**
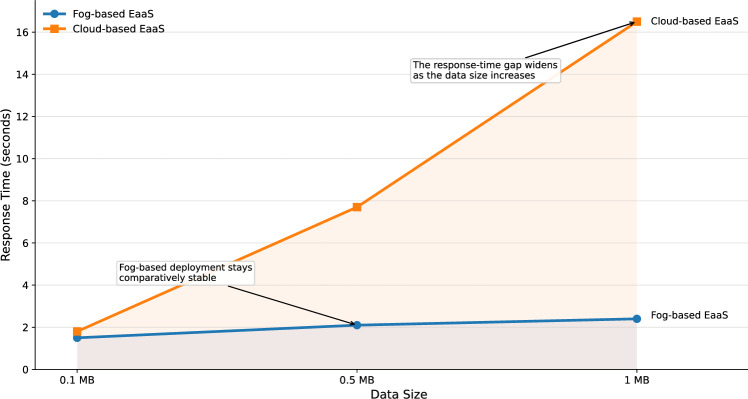
Comparing the average response time for cloud-based and fog-based methods used by the proposed EaaS framework.

For production-oriented studies, average performance should be complemented by tail metrics such as P95 and P99 response time, along with timeout and failed-request rates during scaling events. We therefore make this requirement explicit in the discussion, although the current prototype figures report average values only. Figure 12 compares the response time of cloud-based and fog-based methods in our proposed framework.

After analyzing the data, it becomes apparent that the fog-based deployment outperforms the cloud-based deployment regarding response time. The findings demonstrate a significant advantage for the fog-based deployment, with response time being at least $16\%$ faster in the scenario of having data items of 0.1 MB. This performance gap widens even further to up to 6.8 times faster in the 1 MB scenario. Significantly, as the data size increases, the cloud-based deployment experiences a notable increase in response times. For example, when transitioning from a 0.5 MB to a 1 MB data size scenario, the response time increases more than double, resulting in a slowdown of the services. This suggests that the cloud-based deployment may encounter difficulties in efficiently handling larger data payloads. The difference in performance results between cloud-based and fog-based methods can be attributed to service placement, network distance, and the use of short-lived key-handle caching at the fog side. At the same time, because the current evaluation does not emulate a real WAN path between fog and cloud, these results should be interpreted as controlled prototype results rather than as a complete Internet-scale latency study. Overall, the results demonstrate the efficiency of the fog-based deployment in this particular scenario.

### Operational scope and limitations

The experimental setup uses container-based IoT emulators with constrained CPU and memory instead of physical embedded boards. This choice enables repeatable tests, but it does not fully represent low-power ARM devices or hardware cryptographic accelerators. For the same reason, the present results should be interpreted as prototype-level measurements rather than as final deployment guarantees. A broader validation campaign should include real fog-cloud network emulation, physical IoT boards, and larger tenant mixes.

The current plots emphasize average values. For deployment in production, the platform should also report tail metrics such as P95 and P99 response time, timeout rate, failed-request rate, and behavior during scale-out events. We now state this limitation explicitly so that the interpretation of the current results remains accurate and transparent.

## Conclusion

This paper presented a cloud-native Encryption-as-a-Service (EaaS) platform for Internet of Things (IoT) environments and described its implementation on Kubernetes. The proposed platform supports two deployment models, namely cloud-based and fog-based deployment, and provides encryption, decryption, request handling, and key-management functions through containerized microservices. In addition to the architectural design, the paper clarified the trust boundary of the service workflow, the role of the Key Manager, the protection of long-term keys, and the secure communication assumptions between platform components.

The evaluation focused on three main metrics: processing time, deployment time, and end-to-end response time. The results showed that AES remained the fastest local cryptographic option among the evaluated algorithms because of its low computational cost. However, the proposed EaaS platform provided a more flexible and scalable service model for resource-constrained IoT devices, especially when cryptographic operations were offloaded to dedicated platform components. The results also showed that the fog-based deployment achieved lower response time than the cloud-based deployment in the evaluated prototype setting, with at least $16\%$ improvement for small payloads and up to $6.8\times$ faster response for larger data sizes. In addition, the deployment study showed that scaling the number of replicas increased deployment time in a moderate way, while the Key Manager remained the most resource-intensive component and had the strongest effect on pod readiness time.

Beyond performance, this work also highlighted practical security and deployment considerations for production-oriented EaaS platforms. In particular, the design discussion clarified the key lifecycle, including creation, storage, rotation, revocation, and deletion, and explained the use of protected key storage, audited access, and tenant-aware containment measures. The paper also clarified that namespace separation and RBAC alone are not sufficient for strong multi-tenant isolation and therefore should be combined with network policies, pod security controls, runtime protection, and secure service-to-service communication. These points are important because EaaS is not only a cryptographic engine, but also a distributed service platform that must remain secure under realistic cloud and fog deployment conditions. At the same time, the current evaluation has several limitations. The experiments were conducted in a prototype environment and did not fully emulate wide-area cloud–edge network conditions such as realistic latency, jitter, and bandwidth variation. In addition, the standalone IoT baseline was container-based rather than a physical embedded device, and the current analysis mainly emphasized average timing results. Therefore, the reported results should be interpreted as a prototype-level performance study rather than a complete production benchmark.

Future work will extend the platform and the evaluation in several directions. First, the system should be tested under realistic network emulation and on physical IoT hardware to better capture real deployment behavior. Second, the evaluation should include tail-latency and reliability metrics such as P95, P99, timeout rate, and failed-request rate during scaling events. Third, future versions of the platform can further strengthen supply-chain security, confidential deployment support, and policy-based admission control. Overall, the results indicate that Kubernetes-based EaaS is a promising and practical approach for supporting secure IoT services, and that fog-oriented placement can provide clear latency benefits when low response time is required.

## Data Availability

No datasets were generated or analysed during the current study.
